# ‘Too many mice make no lining for their nest’ – Reasons and effects of parallel governmental structures for disaster risk reduction and climate change adaptation in Southern Africa

**DOI:** 10.4102/jamba.v13i1.1041

**Published:** 2021-06-17

**Authors:** Per Becker, Magnus Hagelsteen, Marcus Abrahamsson

**Affiliations:** 1 Division of Risk Management and Societal Safety, Faculty of Engineering, Lund University, Lund, Sweden; 2 Unit for Environmental Sciences and Management, Faculty of Natural and Agricultural Sciences, North-West University, Potchefstroom, South Africa

**Keywords:** disaster risk reduction, climate change adaptation, DRR, CCA, parallel, governance, Southern Africa, SADC

## Abstract

Many African countries face escalating challenges of increasing disaster risk and anticipated impacts of climate change. Although disaster risk reduction (DRR) and climate change adaptation (CCA) are tightly linked and comprising virtually identical practices in vulnerable countries in Southern Africa, research has identified parallel governance structures across the region. This study applied comparative case study research, based on 27 semi-structured interviews, to investigate the reasons for and effects of such parallel structures for DRR and CCA in Botswana, Mozambique, the Seychelles, Tanzania and Zambia. It revealed overwhelmingly negative effects in terms of unclear mandates and leadership, uncoordinated efforts, duplication of efforts, suboptimal use of resources and competition over resources and control. The study identified both external reasons for the parallel structures, in terms of global or international initiatives or incentives, and internal reasons, with regard to the history and quality of the governance structures. Although the identified negative effects are common to a range of complex nexuses, there is a clear distinction with the DRR–CCA nexus comprising virtually indistinguishable practices in Southern Africa. There is, as such, no practical reason for keeping them apart. The parallel structures for DRR and CCA are instead the result of pervasive institutionalisation across the region, driven by coercive, mimetic and normative pressures coming from both within and abroad. Although much point to the difficulties of changing the studied institutional arrangements, these parallel structures for DRR and CCA must be addressed if the populations in Southern Africa are to enjoy safety and sustainable development.

## Introduction

Although many African countries have seen rapid development in recent decades (UNDP 2019), our oldest inhabited continent is facing mounting challenges in terms of increasing disaster risk and anticipated impacts of climate change (Intergovernmental Panel on Climate Change [IPCC] 2012). The last decades have thus seen a sharp increase in the interest in disaster risk reduction (DRR) and climate change adaptation (CCA) of both African governments (Van Niekerk 2015; Van Niekerk & Coetzee 2012) and the international community (Becker 2014; Schipper 2009).

The temporal coinciding of the emergence and growth of these two policy areas spurred a heated academic debate over the extent to which the two concepts overlap (Kelman & Gaillard 2008; Mercer 2010; Mitchell & Van Aalst 2008; Schipper 2009; Shea 2003). Although influential definitions of CCA also include potential benefits of climate change (IPCC 2014), adapting to the potential negative impacts of climate change is by far the main focus (Satterthwaite et al. 2009), particularly in Southern Africa (Becker, Abrahamsson & Hagelsteen 2013), making CCA more or less a part of DRR in practice (Becker 2014; Mercer 2010; Mitchell & Van Aalst 2008). Moreover, although DRR may include all types of hazards, climate-related hazards (e.g. floods, droughts, wildfires, storms) are associated with the vast majority of disasters triggered by natural phenomena in Southern Africa (Centre for Research on the Epidemiology of Disasters [CRED] 2020). Regardless of what can be described as a general but somewhat strained contemporary agreement of significant overlaps between DRR and CCA, both conceptually and practically, research shows that parallel structures have been created for the two in many countries in Southern Africa (Becker et al. 2013; Nemakonde 2016; Van Niekerk 2015).

Ample studies identify parallel structures in the governance of various sectors in countries across Africa. Although there are studies also suggesting positive aspects of parallel structures in some contexts (Animashaun 2009; Kendie & Guri 2007), most conceive parallel structures in relation to negative effects (Kendie & Guri 2007; Lange 2008; Lund 2006; Nemakonde 2016; Stockmayer 2005; Van Niekerk 2015). However, the effects of parallel structures for the overlapping policy areas of DRR and CCA have not yet attracted much attention, Nemakonde et al. (2021) being one of few exceptions. Furthermore, it is also important to grasp the reasons for establishing parallel structures for DRR and CCA (Becker et al. 2013), which has also so far not attracted much attention in Southern Africa (Nemakonde 2016). Increased knowledge about the effects of parallel structures would provide insight into eventual necessity of making changes to the institutional set-up. In addition, knowledge about reasons for the establishment of parallel structures would provide guidance on where to focus efforts to facilitate such a change.

**FIGURE 1:**
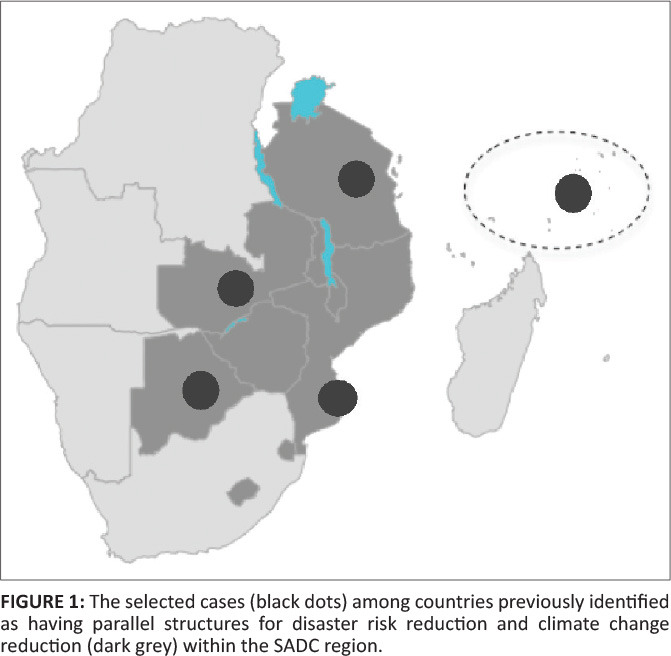
The selected cases (black dots) among countries previously identified as having parallel structures for disaster risk reduction and climate change reduction (dark grey) within the SADC region.

The purpose of this study was therefore to investigate the perceived reasons for and the effects of parallel structures for DRR and CCA in the Southern African Development Community (SADC) region (Figure 1). To meet that purpose, comparative case studies based on qualitative semi-structured interviews with policymakers, directors and technical experts representing government institutions in DRR or CCA were conducted to answer the following research question: *What are the reasons for and the effects of parallel governmental structures for DRR and CCA in Botswana, Mozambique, the Seychelles, Tanzania and Zambia?*

## Methodology

Comparative case study research was deemed suitable to answer the research question (Yin 2003). The aim here is not to arrive at statistical generalisations, but instead analytical generalisations for which case studies have proved suitable (Flyvbjerg 2001). The knowledge developed from the selected cases can thus not be generalised ‘through abstraction and loss of history and context’, but might be transferred to other situations through ‘conscious reflection on similarities and differences between contextual features and historical factors’ (Greenwood & Levin 2007:70).

To study the reasons for and the effects of parallel structures for DRR and CCA in Southern Africa, five cases were selected from a pool of nine countries that Becker et al. (2013) had identified as exhibiting variations of such organisational arrangements (Figure 1).

Resource restrictions allowed for visits to five countries, which were purposefully selected to include governance structures in countries categorised as having low (Mozambique and Tanzania), medium (Zambia) and high human development (Botswana and the Seychelles).

Data were collected through 27 qualitative semi-structured interviews with policymakers, directors and technical experts involved in DRR or CCA in the five countries. The participants were selected purposefully in an attempt to cover these three groups of actors in each country. The five countries were purposefully selected to include countries with different levels of development, operationalised as their current Human Development Index (HDI) (United Nations Development Programme [UNDP] 2020). The Seychelles has the highest HDI in Africa and sits just below the border to very high human development (rank 67). Botswana is categorised as having high human development (rank 100) and Zambia as having medium human development (rank 146). Tanzania is among the top of the countries categorised as having low human development (rank 163), while Mozambique is among the bottom of the countries in that category (rank 181) and the least developed country in the SADC region.

Qualitative interviews provided opportunities to attain in-depth information based on the participants’ experiences and perceptions (Trost 2005) and to follow up, probe or confirm information (Brinkmann & Kvale 2015:132–133). Semi-structured interviews were preferable considering the participants’ busy schedules, because they are relatively less time-consuming than completely unstructured interviews (Bernard 2006). The interviews were conducted between January and May 2014, taking on average 45 min each.

Participants were purposefully selected (Bernard 2006) for their position, organisational affiliation and involvement in DRR or CCA. The selection was initiated based on a few already identified informants from the authors’ professional networks, and snowballing was used to identify further informants (Hennink, Hutter & Bailey 2011:100). Six participants were interviewed in Botswana, six in Mozambique, six in the Seychelles, five in Tanzania and four in Zambia. Six of the participants were women and 21 were men, mirroring the male dominance of these policy areas in the studied countries. The participants included directors, deputy directors, head of departments, divisions or units, programme officers, national coordinators, focal points, advisors or experts in DRR and CCA at various governmental organisations and committees. The following were specifically targeted: national disaster management offices, climate change secretariat, meteorological services and departments of risk and disaster management, meteorology, forestry and energy. Of the 27 participants, 16 represented the policy area DRR and 11 participants represented CCA. Several participants had experience of working with the United Nations. All the interviews were conducted in English with the help of an interview guide in three parts (Table 1).

**TABLE 1:** Interview guide.

Part	Question
Part 1	What is your position? What is your background?
Part 2	What do you think are the reasons for establishing separate structures for disaster risk reduction and climate change adaptation in your country? What do you think are the effects of establishing separate structures for disaster risk reduction and climate change adaptation?
Part 3	Summary of key issues. Anything else to add? Showing appreciation and asking how the participant experienced the interview.

The interviews were recorded with the permission of the participants to ensure consistency and accuracy (Bernard 2006:227) and transcribed in verbatim. Field notes were taken during the interviews to document impressions and make connections within and between themes (Trost 2005). The transcribed interviews were hand-coded on paper to enable the authors to ‘touch the data’ (Saldaña 2015:21–22). Codes were discussed and adjusted between the authors during the analysis process, because data could be interpreted from different angles and perspectives.

### Limitations

The study is based on data from interviews conducted in 2014. The studied policy areas undergo constant change, and one should note that the findings of the study represent the situation at the time of data collection.

## Findings

Although the focus of the study and the conducted interviews is on the reasons for and the effects of parallel structures for DRR and CCA, the results also turned out to include ideas concerning solutions to perceived negative effects of parallel structures. This results chapter is thus divided into these three parts, each presenting the main themes emerging from the analysis of the empirical material.

### Reasons for parallel structures

The first part of the results from the study concerns the reasons for establishing the parallel structures for DRR and CCA existing in the five countries at the time of data collection. Two main themes emerged from the study, and also voices pointing out that there are no real parallel structures in their countries.

#### External reasons: Global or international initiatives or incentives

The first and the most common account of the reason behind establishing parallel structures for DRR and CCA in the studied countries was concerned with global or international initiatives. These initiatives consist of incentives and pressures. Such external pressures on the governance structures of the five countries were mentioned in different ways by around half of the participants. This is regardless of the governance structures for CCA having an evident and exclusive focus on reducing climate-related risks in all five studied countries: not on attempting to reap any potential benefits of a changing climate. The participants explained the establishment of new structures for CCA, even when there were structures in place for DRR, partly in terms of an explicitly or implicitly experienced condition for accessing particular international funding streams (e.g. the Global Environment Facility). In the words of one participant:

‘They have to create something, otherwise they wouldn’t get anything from the international community’. (DRR representative 4)

Such accounts thus framed the establishment of parallel structures for DRR and CCA in terms of the countries’ governments simply responding to new emerging global opportunities by setting up structures that they thought would facilitate access to additional resources. However, these rather externally oriented explanations did not stop there. In addition to anticipated increased access to resources, the participants also suggested that the establishment of parallel structures could also be traced back to the organisation of these issues within the international community itself. Hence, it implies that the issues of DRR and CCA were also largely addressed by parallel structures on the global level and then mirrored on the national level. For instance:

‘Maybe it comes from the UN. They are separate at the UN, and it means that even when you go to the countries they are also separate’. (DRR representative 10)

Finally, there were also participants linking external and internal reasons when explicitly pointing out that the new opportunities to access additional financial resources were also instigating or exacerbating internal competition between different parts of government. For example:

‘there is a better way to deal with that, but it is this international influence that creates higher costs for government who is competing for resources’. (CCA representative 5)

Such competition is further elaborated on as an effect of parallel structures below, but was also in a sense a reason for establishing parallel structures. This since parts of government not previously engaged in reducing climate-related risks in the studied countries managed to expand their mandate to obtain control over the resources in the process.

#### Internal reasons: The history and quality of governance structures

Around a quarter of the participants provided more internally oriented reasons for the establishment of parallel structures for DRR and CCA. These explanations partly revolved around the history of the national governance structures for DRR, which all grew out of the exclusively disaster response-oriented structures of the past. Even if their mandates had explicitly included proactive risk reduction for years before the time of data collection, these participants framed the DRR structures either as inappropriate for more proactive reduction of climate-related risks, or as simply failing to shoulder the responsibility to engage in it. These two arguments come together in the account of one participant:

‘I think it was based on the establishment of the disaster management department. […] It was purely a unit for coordination of relief. So, they did not integrate the DRR concept and the development issues on DRR’. (DRR representative 1)

Another but related line of argument was less focussed on the history of the governance structures for DRR and CCA, and more focussed on their current qualities. Here, other participants instead expressed an internal reason for establishing parallel structures based on a difference in what drove the two policy areas in the studied countries at that time, where DRR was seen as driven by local needs for risk reduction materialising as preventable disaster consequences, whereas CCA was seen as driven largely by global concerns materialising as anticipated scenarios of escalating disaster risk. In the words of one participant:

‘So climate change is driven from international policy […] while DRM was driven from reality on the ground’. (CCA representative 11)

#### Not parallel structures

It is also important to note that there were a couple of participants in the Seychelles and Mozambique, who maintained that there were no real parallel structures for DRR and CCA in their countries. In Mozambique, there is to some extent an organisational overlap between the two areas, with the organisation responsible for DRR also coordinating adaptation. Participants in the Seychelles pointed out that although there were different departments focussing on DRR and CCA at the time of the interviews, they both resided under and were coordinated by the same ministry.

### Effects of parallel structures

The next part of the results concerns the effects of having parallel structures for DRR and CCA from the perspectives of the participants. This part comprises six main themes, five of which elaborate on different but connected negative effects, whereas one theme is suggesting some positive effects.

#### Unclear mandates and leadership

The first main theme concerning the effects of having parallel structures for DRR and CCA at the time of the interviews focussed on unclear mandates and leadership. Around a quarter of the participants included such issues in their narratives. They mentioned confusion regarding mandates and responsibilities in different ways, as well as how the two governance structures were to interact with each other, which undermines effective reduction of climate-related risks. In the words of one participant:

‘You know, you also bring confusion in terms of the lack of understanding on the respective responsibilities. What exactly do we need to basically do under this? Maybe what you’re doing under this should be input under the other one’. (DRR representative 8)

These challenges were also connected to confusion concerning leadership, where the participants expressed in different ways how parallel governance structures for DRR and CCA entailed difficulties in knowing who was in charge of different activities. Such unclear leadership was seen as detrimental for the reduction of climate-related risks. For instance:

‘So it becomes a little bit confusing. […] Okay, so who leads what efforts? So the leadership issue becomes you know, compromised’. (CCA representative 5)

#### Uncoordinated efforts

The second theme in the participants’ accounts concerned a significant lack of coordination between DRR and CCA at the time of data collection. Having two parallel governance structures engaged in more or less identical, or at least significantly overlapping, activities, one example being efforts to reduce coastal flooding problems, was pointed out by a quarter of the participants as resulting in uncoordinated efforts to reach the same overarching objectives. This was phrased in various ways by the different participants, and quotes from the following two can serve as examples:

‘Because of the set-up, you miss the coordination’. (CCA representative 1)

‘So there tends to be a coordination gap as to who do what, when and where’. (CCA representative 10)

#### Duplication of efforts

The next main theme is tightly connected to uncoordinated, but more specific, efforts. This theme concerned a problem with duplication of efforts in the activities of the two parallel governance structures for DRR and CCA, where different actors engage in activities addressing the same thing without considering, or even being aware of, each other’s activities. This was stated more or less explicitly by a quarter of the participants. For instance:

‘Where we are now, there’s a possible chance … a chance of duplicating efforts because we have the disaster management people planning and strategising on their own, while at the same time we have climate change people also coming up with their own structures, and trying to implement them’. (CCA representative 7)

‘I think there is danger that we could duplicate the efforts. If we don’t work closely, we might be dealing with one item, the two offices’. (DRR representative)

#### Suboptimal use of resources

Lack of coordination and duplication of efforts are linked to a fourth theme emerging in the narratives of around a fifth of the participants, which specified that the parallel governance structures for DRR and CCA resulted in less efficient use of resources at the time of the data collection. Some of these participants explicitly described in various ways how resources were wasted by maintaining the two formal organisations responsible for coordinating DRR and CCA. In the words of one participant:

‘I also see the challenges related to costs, in terms of cost of running these two divisions’. (DRR representative 2)

It is not only the organisational costs that the participants pointed out as problematic, but also the wasteful operational costs of the uncoordinated and often duplicated efforts referred to above. They voiced significant frustration over this, as resources are always scarce and there are other ways of working. This is summarised well by one participant:

‘So, over time when you calculate how much you have spent on the same thing … You know the costs could have been prevented. So it is an expensive venture. And, of course, there is a better way to deal with that, but it is this international influence that creates higher costs for government, which is competing for resources’. (CCA representative 5)

#### Competition for resources and control

The quote above is not only demonstrating thoughts on suboptimal use of resources, but also regarding competition between the parallel governance structures for DRR and CCA. This theme was further elaborated on by around a fifth of the participants, who described in different ways the internal struggles between the different governmental offices on the national level to access resources and exert control. For instance:

‘So yeah, at the country level it becomes a battle for control in who does what, with higher authority over the others. […] Departments want big portfolios so they want to add on some of these interesting ideas. You know bigger budget, more resources, so naturally at the country level people begin to ask for more. So that gets challenges’. (CCA representative 5)

Interestingly enough, participants on both sides of the divide between the governance structures for DRR and CCA expressed opinions concerning the other side having easier access to resources, with participants representing the former stating that CCA attracts massive funding and participants representing the latter stating that it was easier for DRR to get resources allocated: often in relation to the administrative location of the particular office. For instance:

‘It’s easier for them [*DRR system*] to get the money through the office of the president’. (CCA representative 6)

#### Positive effects

In addition to the five themes pointing out different but connected negative effects of parallel structures for DRR and CCA, there were also a couple of participants describing a few positive effects. Although these participants also contributed to the themes focussing on the negative effects above, they suggested that having parallel governance structures could increase the focus on reducing climate-related risks simply by having more people advocating for it. They also suggested that it could result in more financial resources being allocated to these pertinent issues.

### Solutions to the perceived negative effects of parallel structures

In addition to the reasons for and the effects of parallel governance structures for DRR and CCA explicitly addressed by dedicated themes in the interview guide, the conversations with some participants developed to involve also some ideas for solutions to address the effects of the parallel structures. Two main themes emerged here.

#### Joint planning and projects

The first theme connected to solutions for the effects of the established parallel structures for DRR and CCA in the studied countries was concerned with joint planning and projects. Although this was only mentioned by a few participants, they described in different ways how they either could or already did actively address the negative effects. Here, they mentioned mainly either planning together, or implementing actual projects together. For instance:

‘So, we are trying to see how best we can pull together resources and together come up with the common initiatives’. (DRR representative)

It is important to note that the participants, stating that they were actively trying to tackle the problems of having parallel governance structures, also explicitly acknowledged this way that these attempts were limited to particular projects. They further stated that each side had lots of more projects without the other side being involved.

#### Connecting or merging

If the solution based on joint planning and projects would try to address the effects of the parallel governance structures, a few participants also proposed in different ways to address the parallel structures altogether. Suggestions were made to either connect the governance structures for DRR and CCA in a way to overcome the negative effects of the parallelism, or to merge both DRR and CCA into one governance structure. One participant even stated that there was a process initiated in that country to just do that:

‘… there is a process now to see how the two can be brought together, because by implication they have to respect each other, you cannot have them in silos’. (CCA representative 5)

## Discussion

It is evident that the countries in Southern Africa are particularly at risk in terms of increasing disaster risk and anticipated impacts of climate change (IPCC 2012; Van Niekerk 2015). Yet, the results of this study demonstrate a range of substantial and interconnected problems with the almost universal set-up of parallel governance structures for DRR and CCA across the region, which completely overshadow the little potential positive effects mentioned by much fewer respondents. The results point out the issues of unclear mandates and leadership and uncoordinated efforts, which lead to duplication of efforts and inefficient use of the already scarce resources available to reduce escalating climate-related risks. Such problems are not unknown in established theory, but must be addressed if the populations in Southern Africa are to enjoy safety and sustainable development (Nemakonde 2016). Leadership is commonly identified as crucial (International Federation of Red Cross and Red Crescent Societies [IFRC] 2015; Lopes & Theisohn 2003; UNDRR & Coppola 2019), although Mitchell (2006:236) reminds us that it is not a panacea to all ills. Lack of coordination is almost always identified as a primary Achilles heel of both DRR and CCA (e.g. Artur & Hilhorst 2017; Twigg 2004; Wamsler 2014:167–170), as well as of international development cooperation in general (Baranyi & Desrosiers 2012; Bebbington & Farrigon 1992:55–56), and duplication and inefficient use of resources are common problems (Anderson & Holcombe 2013:308; Nemakonde 2016; Quarantelli 2003:220). The results also include competition between actors, which is also a well-known problem in situations with many actors (Artur & Hilhorst 2017:7; Handmer & Dovers 2007:132; Mitchell & Van Aalst 2008). These problems of parallel governance structures for DRR and CCA thus allude to a traditional Shona proverb, which freely translated says ‘Too many mice make no lining for their nest’,^1^ hence the title of this article.

Regardless of the effects of parallel structures for DRR and CCA being common problems between the parts of a whole range of identified nexuses – such as the disaster –development (Collins 2009), development – climate (Davidson et al. 2003) and humanitarian – DRR nexuses (United Nations Office for the Coordination of Humanitarian Affairs [OCHA] 2018:73) – it is worth noting a significant distinction from most of them. Such influential conceptualisations of nexuses are constructed to highlight the interdependencies between separate, yet interconnected sets of activities, which if seen as a whole would increase the chances of success for each of them and for some overarching goal. Although there may be some overlaps between these sets of activities, there are clear differences in purpose, functions and activities. This is in sharp contrast to the DRR – CCA nexus identifiable in the results, with both parts being virtually indistinguishable in these regards. They are thus not separate and interconnected – like in the other nexuses – but actually more or less the same in all important aspects. Despite the prevailing academic debate over the conceptual overlap or separation of DRR and CCA (Islam, Chu & Smart 2020; Kelman & Gaillard 2008; Mercer 2010; Mitchell & Van Aalst 2008; Schipper 2009; Shea 2003), the results contain little that warrants treating them as distinct from each other.

As there are no real differences in neither purpose, nor functions, nor actual activities between DRR and CCA in the studied countries in Southern Africa, there must be other reasons behind the parallel governance structures. The results suggest a combination of external and internal reasons, where the former comprise global or international initiatives or incentives, while the latter involve the history and quality of the governance structures. It is also striking to note the astounding similarity in the parallel governance structures at the time of data collection, with only the Seychelles hosting both offices in the same ministry and Mozambique concentrating more of CCA in their competent authority for DRR. However, it is important to consider that the Seychelles is a very small country, with a small government. Although this may facilitate coordination, as suggested by some respondents, it is still noteworthy to find parallel offices there. This is, however, not the first time researchers identify such patterns in an organisational field. Meyer and Rowan (1977) and Dimaggio and Powell (1983) refer to this phenomenon as institutional isomorphism in their two seminal papers launching new institutionalism as a theoretical perspective. This perspective is particularly useful when trying to understand why parallel governance structures for DRR and CCA are developed and maintained despite their overwhelmingly negative effects.

New institutionalism effectively debunks the myth that organisations are structured and functioning only for rational goal-oriented efficiency (Scott 2014). Regardless how common such ideas still are in society, this perspective demonstrates that organisations are also structured by institutional rules (DiMaggio & Powell 1991; Scott 2014). Dimaggio and Powell (1983:147) describe isomorphism as ‘the result of processes that make organizations more similar without necessarily making them more efficient’, which can be coercive, mimetic or normative. Going back to the results, it is evident that the governments experienced pressure to establish a new governance structure for CCA not to miss opportunities to tap into emerging funding streams. Although it was obviously their sovereign choice, it is difficult not to see the fundamental aspects of coercion behind such decisions, especially, as all studied countries are either among the least developed, the most vulnerable to escalating climate-related risks, or both. This is fully in line with Dimaggio and Powell’s (1983) notion of coercive processes behind institutional isomorphism, which have been shown to play crucial roles in a range of other organisational fields in Africa (e.g. Home 2020:73; Maroun & Van Zijl 2016). The results also include explicit signs of mimetic processes, which are instead rooted in uncertainty and entail copying the organisational structure of someone deemed more competent (DiMaggio & Powell 1983). It is, in other words, not only the threat of losing out on much needed funding that pushed governments to establish parallel structures for DRR and CCA, but also them responding to rapid global change and escalating uncertainty by simply mimicking global structures. It is also likely that such mimetic processes intensify when other countries that are considered more competent in the particular field also establish parallel structures, especially because other studies demonstrate such processes between actors in Africa (e.g. Maroun & Van Zijl 2016; Masocha & Fatoki 2018). Mimetic processes have also been suggested to influence both disaster response-oriented organisations and their increasing focus on DRR in recent decades (Prakash et al. 2020). This is linked to Dimaggio and Powell’s (1983) last type of processes behind institutional isomorphism; normative processes associated with professionalisation and professional culture. The results suggest an important role of professional cultures in the establishment of parallel governance structures for DRR and CCA in at least two ways: firstly in the sense of emphasising the disaster response-oriented history of the governance structures for DRR, which still determined much of their current professional cultures according to some participants, and secondly in suggesting innate differences in professional cultures between DRR and CCA, in which the former is seen as hands-on and driven by local needs and the latter as more policy oriented and driven by global concerns. Although it is difficult to ascertain the relative importance of these three types of processes behind the studied institutional isomorphism, it is when such institutional pressures align that their combined force is most formidable (Scott 2014:70–71).

Considering the overwhelmingly negative effects of the parallel structures for DRR and CCA, as well as the few constructive voices advocating connecting or merging them, it is interesting to contemplate the propensity for actual change of the governance structures. Pearson (2011:16) calls this ‘change readiness’ and argues convincingly for the importance of ‘conditions, attitudes and resources, at all levels in a system, needed for change to happen’. There are, in other words, plenty of different factors that need to align for change to transpire under present coercive, mimetic and normative pressures; not only the good intentions of a few people. Locating where there is already readiness for change in the governance structures is also fundamentally important for any purposeful activities to cultivate it (Pearson 2011:17). Without substantial readiness for change, the parallel structures will remain for sure.

It is also interesting and important to consider what would happen if DRR and CCA do merge into one governance structure. The present study is in no position for any authoritative suggestions concerning this. Additional research is needed for that. However, one way of pursuing such research agenda would be to study cases with such joint structures to see if they do attract more or less funding in total, if they are more successful in coordinating multiple activities and so on. It would also be interesting to follow up the cases in which participants explicitly stated that processes had started to improve the institutional set-up at the time of data collection.

## Conclusion

The study reveals that there were a couple of interacting reasons for, and several and overwhelmingly negative effects of, the parallel governance structures for DRR and CCA in the studied countries in Southern Africa.

Although the identified negative effects are common to a range of complex nexuses, there is a clear distinction with the DRR–CCA nexus comprising virtually indistinguishable practices in Southern Africa. There is, as such, no practical reason for keeping them apart. The parallel structures for DRR and CCA are instead the result of pervasive institutionalisation across the region, driven by coercive, mimetic and normative pressures coming from both within and abroad. While most point to the difficulties of changing the studied institutional arrangements, these parallel structures for DRR and CCA must be addressed if the populations in Southern Africa are to enjoy safety and sustainable development in future.

## Footnotes

1 Similar to the English proverb: ‘Too many cooks spoil the broth’.
